# A Peripheral Ameloblastic Fibro-Odontoma in a 3-Year-Old Girl: Case Report, Immunohistochemical Analysis, and Literature Review

**DOI:** 10.1155/2014/321671

**Published:** 2014-06-30

**Authors:** Yi-Chun Lin, Hsiu-Ming Hsu, Chiang-Shin Liu, Kuo Yuan

**Affiliations:** ^1^Division of Pediatric Dentistry, Department of Stomatology, National Cheng Kung University Hospital, Tainan 704, Taiwan; ^2^Institute of Oral Medicine, College of Medicine, National Cheng Kung University, Tainan 701, Taiwan; ^3^Department of Pathology, National Cheng Kung University Hospital, Tainan 704, Taiwan

## Abstract

Ameloblastic fibro-odontoma (AFO) predominantly occurs in the jaw bones of children and young adults. Extraosseous AFO is extremely rare. We describe a peripheral ameloblastic fibro-odontoma in the maxillary gingiva of a 3-year-old girl. The clinical appearance resembled fiery red reactive gingival lesions. The histopathological examination of the excised lesion showed small islands and cords of odontogenic epithelium with cellular myxoid stroma in the subepithelial tissue. The mass contained calcified material and an enamel-like deposit. Many small blood vessels appeared in the connective tissue surrounding the odontogenic epithelium. The immunohistochemical assays showed strong reactivity for amelogenin, *β*-catenin, CD44, and CD31 in the tissue sections. There was no recurrence after the 1-year follow-up. Because this lesion clinically resembles other nonneoplastic lesions and is very rare in gingiva, establishing a correct diagnosis is achieved only based on specific histological characteristics. Conservative excision of the tumor is the treatment of choice.

## 1. Introduction

Ameloblastic fibro-odontoma (AFO) is “a rare odontogenic tumor with the histopathological features of an ameloblastic fibroma (AF) in conjunction with the presence of dental hard tissue” [1]. The soft tissue constituent of the tumor is composed of epithelial cords and small isles of odontogenic epithelium associated with a primitive-appearing myxoid connective tissue that appears like the dental papilla. The hard tissue constituent consists of foci of dentin and enamel with varying degrees of mineralization [2]. It is usually slow developing and is usually seen in young persons with a mean age between 8 and 12 years [1, 3]. A majority of AFO is intraosseous and is associated with unerupted teeth. It is reported that approximately 80% of the lesions were located in the posterior area of the jaws, and most (58%) were in the posterior mandible [3]. Peripheral (extraosseous, soft tissue) AF or AFO arising in gingival tissue is even rarer than their intraosseous counterparts [4–6]. Although molecular alternations are reported in different types of odontogenic tumors, their pathogenesis remains to be elucidated [7, 8]. Based on the fact that (i) previous studies have demonstrated that amelogenin, *β*-catenin, and CD44 are related to odontogenic tumors [9–13], (ii) this reported lesion presented with fiery red appearance and abundant small blood vessels in histological section, and (iii) CD31 (platelet endothelial cell adhesion molecule-1 (PECAM-1)) is the most well-known cell marker for endothelial cell [14], we did an immunohistochemistry of amelogenin, *β*-catenin, CD44, and CD31 for the tissue sections of this case to obtain cellular and molecular information.

## 2. Case Presentation

**Figure 1:**
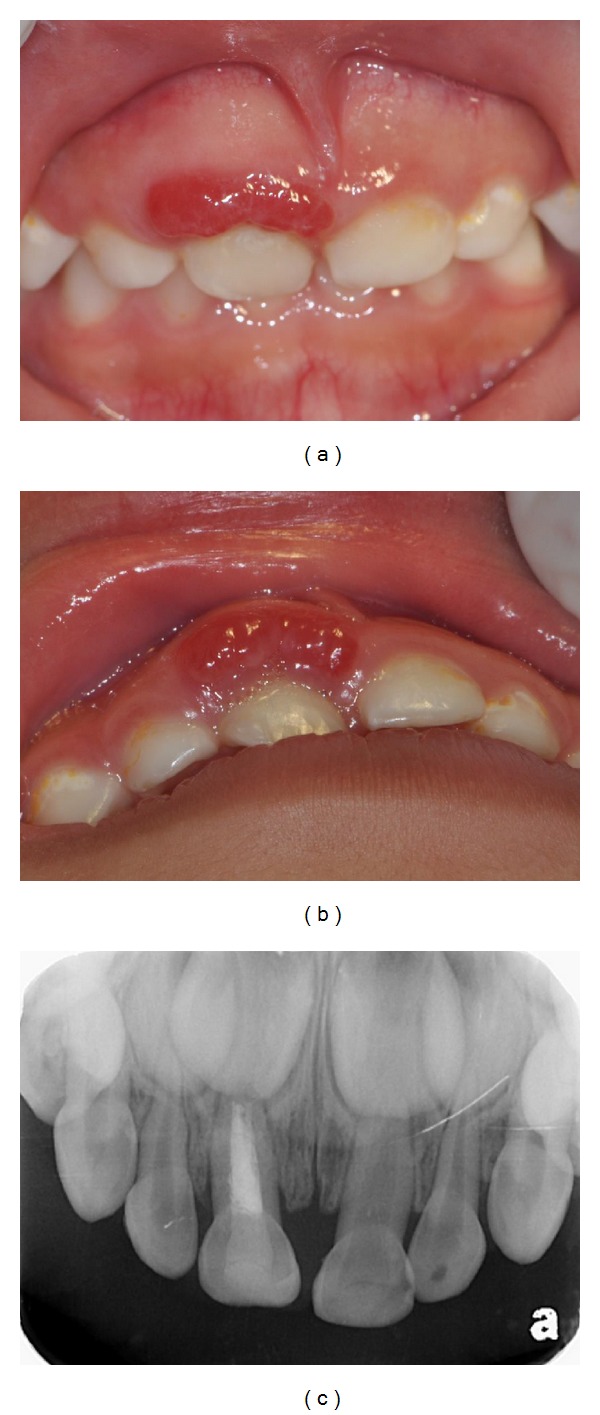
Clinical and radiographic appearances. (a) Frontal view: a fiery red gingival lesion on the labial side of the primary maxillary right central incisor. (b) Occlusal view: the maxillary right primary central incisor was a little palatally displaced. (c) A pulpectomy and restoration had been done for the primary maxillary right central incisor at a local dental clinic. There was no observable radiopaque abnormality in the periapical radiograph.

**Figure 2:**
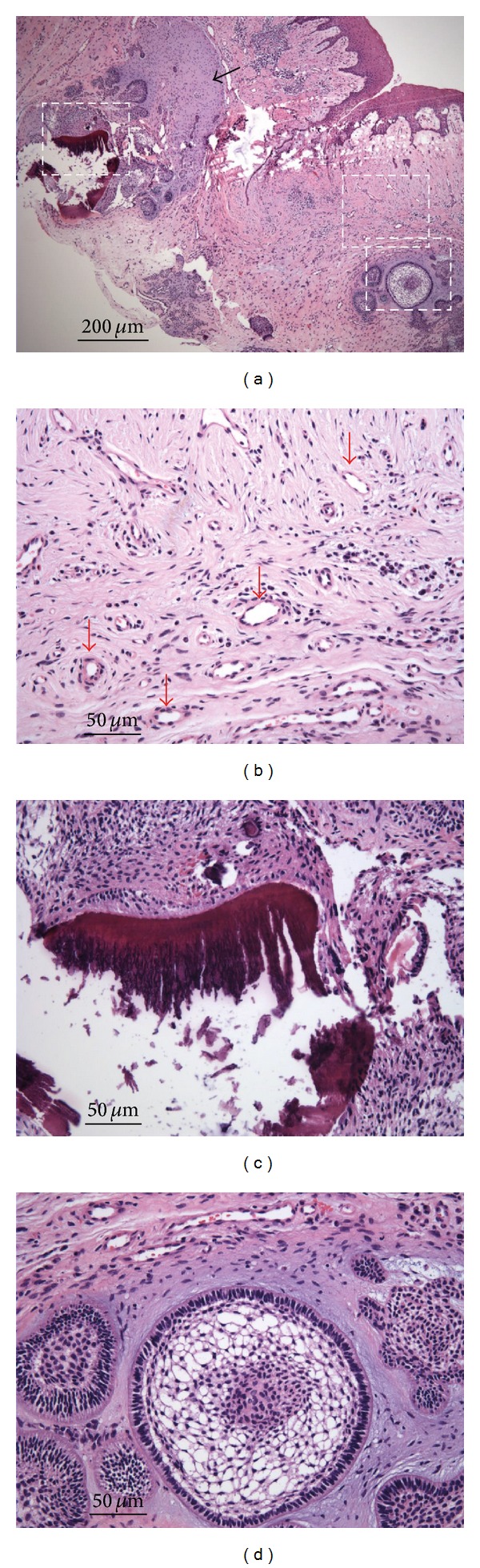
Hematoxylin and eosin staining for the gingival lesion. (a) The black arrow indicates a dental papilla-like tissue. (b) The red arrows denote abundant small blood vessels. (c) A large mineralized matrix appears like enamel or dentin structures. (d) There are several islands of odontogenic epithelium. One of them clearly exhibits enamel organ differentiation. Panels (b), (c), and (d) are magnifications (×4) of the white squares of panel (a). Scale bar = 200 *μ*m for panel (a) and 50 *μ*m for panels (b), (c), and (d).

**Figure 3:**
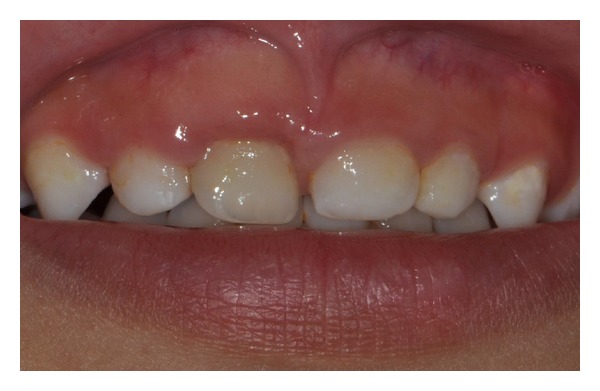
Clinical appearance 1 year after excisional biopsy for the gingival lesion.

A three-year-seven-month-old girl was referred to the Pediatric Dentistry Section, Department of Stomatology, National Cheng Kung University Hospital, to have a swelling and erythematous mass on the facial gingiva of the primary maxillary right central incisor evaluated. According to her parents' statements, the eruption time of this tooth was the latest of all her upper anterior teeth. Immediately after the eruption, it was positioned more palatally than the adjacent incisors. A small, white, tooth-like substance existed on the facial gingiva of this tooth. Then the tooth-like substance spontaneously exfoliated. About 1.5 years ago, the mother became aware of her daughter's gingival mass. She could not remember that her daughter had undergone any trauma. They went to a local dental clinic for evaluation. The dentist performed endodontic treatment for the central incisor because he found caries and speculated that the gingival swelling had an endodontic origin. Five months later, they went to the same clinic because the lesion became larger. The dentist decided to refer the child to our hospital. During her initial examination in our hospital, we noticed a fiery red gingival mass that neither bled nor felt palpation pain (Figure 1(a)). The probing depths of the tooth were within normal range. The tooth was a little palatally displaced but not mobile (Figure 1(b)). The periapical radiograph revealed no periapical lesion, root resorption, or radiopaque abnormality (Figure 1(c)). Our initial but tentative diagnosis was a pyogenic granuloma. After the lesion had been locally debrided and irrigated with 0.2% chlorhexidine, the patient's mother was instructed in oral hygiene techniques. There was no improvement in the patient's condition after 2 weeks of follow-up. After we had consulted with periodontics and oral-maxillofacial surgery specialists, we suggested an excisional biopsy under general anesthesia for the patient. After we had obtained the consent of the parents, we removed the mass using surgical scalpels; bleeding was stopped using electrocautery in the operating room. Amoxicillin and acetaminophen were prescribed for the patient. The surgical specimen was sent to National Cheng Kung University Hospital's Pathology Department for evaluation. The gross specimen measured 10 × 5 × 2 mm. A microscopic examination revealed a small island and cords of odontogenic epithelium in a loose and primitive-appearing connective tissue that resembled dental papilla. The mass contained calcified material and an enamel-like deposit. Abundant small blood vessels surrounded the odontogenic epithelium (Figure 2). The pathological diagnosis was an ameloblastic fibro-odontoma. A one-year postoperative follow-up revealed no evidence of recurrence, but only a mild gingival inflammation (Figure 3). The patient's parents were advised to bring her back for continuous follow-ups and to conscientiously provide her with good oral hygiene.

## 3. Immunohistochemical Assay

**Figure 4:**
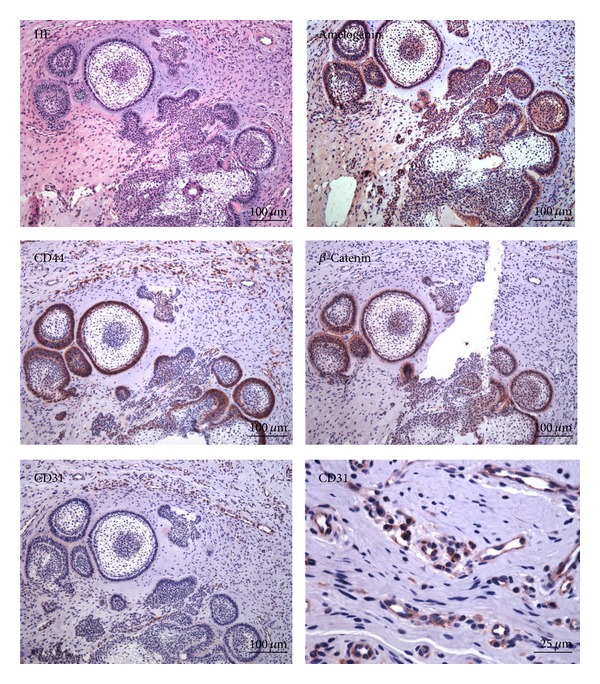
Immunohistochemistry for amelogenin, *β*-catenin, CD31, and CD44. The stainings showed that amelogenin, *β*-catenin, and CD44 were strongly expressed in the enamel epithelial cells. Some of the stromal cells, leukocytes, and endothelial cells were also positive for CD44 staining. CD31 staining appeared in the endothelial cells of the tumor stroma, especially in the area adjacent to the enamel epithelium. Scale bar = 100 *μ*m for all panels but the bottom right (25 *μ*m).

An informed consent was obtained from the patient's parents. They agreed to let us analyze their daughter's specimen using immunohistochemical (IHC) stainings. We purchased the primary antibodies for the amelogenin (Abcam, Cambridge, UK), *β*-catenin (EMD Millipore, Billerica, MA, USA), CD44 (Neomarkers-Labvision, Fremont, CA, USA), and CD31 (Abcam) proteins. Isotype immunoglobulins (Abcam) were used to replace primary antibodies as negative control. The slides were counterstained with Mayer hematoxylin and photographed using an optical microscope (BX61; Olympus, Tokyo, Japan) equipped with an imaging system (Stream; Olympus). The IHC results showed that amelogenin was strongly expressed in enamel epithelium. The calcified matrix in the tissue sections was also positive for amelogenin staining. The enamel epithelial cells were strongly immunoreactive for *β*-catenin and CD44. The subcellular localizations for *β*-catenin were on the cell surface and in the nucleus, whereas CD44 was predominantly on the cell surface. Some of the stromal cells, leukocytes, and endothelial cells were also positive for CD44 staining. CD31 staining confirmed that there were abundant small blood vessels in the tumor stroma, especially in the area adjacent to the enamel epithelium (Figure 4).

## 4. Discussion

Regarding histogenesis, it is still controversial for the relationship between AF, ameloblastic fibrodentinoma (AFD), AFO, and complex odontoma. Some pathologists consider them as separate entities. Others regard them as sequential stages beginning from AF at one extreme and complex odontoma at the other extreme with AFD and AFO in the intermediate stages. AFD is defined as a neoplasm similar to AF that concurrently shows formation of dentin or dentinoid [20]. The difference between AFD and AFO is the existence of enamel or enamel-like structure in the latter entity. The peripheral types of mixed odontogenic tumors are much rarer than their intraosseous counterparts [1–6]. We have summarized the previously reported cases of peripheral AF, AFD, and AFO, together with the current case in Table 1. The age range is 2.5 to 51 years. More female cases are reported than male ones. The reported extraosseous tumors do not appear in the oral mucosa other than gingiva. Compared to their intraosseous counterparts, peripheral tumors are more located in maxillary arch anterior to first molars. Their clinical manifestations resemble much those of localized reactive gingival lesions [4–6]. Localized reactive hyperplastic lesions in gingiva can be classified into 4 subcategories: peripheral ossifying fibroma, peripheral giant cell granuloma, pyogenic granuloma, and focal fibrous hyperplasia [21, 22]. Two additional pathologies, peripheral ameloblastoma and calcifying epithelial odontogenic tumor, should also be distinguished [4]. The definitive differentiations between these clinically similar diseases depend on their microscopic findings.

Our clinical diagnosis for this case was pyogenic granuloma because of its erythematous appearance. It is well recognized that one major histopathological characteristic of pyogenic granuloma is the lesion's numerous capillaries. In our patient, numerous small blood vessels were distributed near the odontogenic epithelium in the tissue sections. There may be two reasons for the high vascularization of this lesion. First, a gingival lesion is subject more than its intraosseous counterpart to constant irritation from plaque, calculus, food impaction, and low-grade trauma. Second, there are several indications that amelogenin protein, highly expressed in the odontogenic epithelium of this lesion, is actually a proangiogenic molecule [23]. The odontogenic epithelium in our patient also expressed high levels of CD44 and *β*-catenin. CD44, a hyaluronic acid receptor, is one of the most commonly studied surface markers, which is expressed by almost every tumor cell [24]. Studies have reported that CD44 is highly expressed in ameloblastomas [13]. *β*-catenin is an important regulator for telomerase activity, which is pivotal in stem cells and cancer because it controls telomere length. Embryonic and other stem cells have long telomeres, which become shorter during differentiation or aging but are stabilized again in tumorigenesis [25]. A genetic engineering study showed that mouse tooth buds expressing stabilized *β*-catenin in oral epithelium give rise to odontoma-like structures containing dozens of malformed teeth [26]. Clinically, it has been revealed that *β*-catenin is highly expressed in odontogenic tumors: ameloblastoma, odontogenic carcinoma, and benign odontomas [11, 12]. The IHC staining implied that CD44 and *β*-catenin might be involved in the pathogenesis of this lesion. Based on the existing literature, amelogenin secreted by the odontogenic epithelium may contribute to the red appearance of the lesion.

The standard treatment for intraosseous AF and AFO is conservative surgery with enucleation and a close clinical follow-up [3]. The recurrence rate of intraosseous AFO is uncommon: less than 8% [3]. Because peripheral AFO is so rare, there is no published study that estimates its recurrence rate after surgical excision. Most studies suggest that conservative excision of the tumor with minimal but adequate margins is the treatment of choice [4, 6].

## 5. Conclusion

We report a rare case of gingival AFO with the classic microscopic features of its intraosseous counterpart. Because peripheral AFOs clinically resemble other neoplastic and nonneoplastic lesions in the gingiva, establishing a correct diagnosis is difficult and is possible only based on a histological distinction. It is plausible that amelogenin expressed by the enamel epithelium contributes to the fiery red phenotype. It appears that the peripheral AFO should be initially treated by conservative surgical therapy and the patient should be clinically followed up for recurrence.

## Figures and Tables

**Table 1:** Reported cases of peripheral ameloblastic fibroma (AF), ameloblastic fibrodentinoma (AFD), and ameloblastic fibro-odontoma (AFO).

Author/year [reference]	Gender/age	Localization	Clinical diagnosis	Pathological diagnosis
Kusama et al./1998 [5]	Female/40 years old	Buccal gingiva of lower right premolars	Fibrous epulis	AF
Abughazaleh et al./2008 [6]	Female/3 years old	Labial gingiva on upper right primary lateral incisor	Pyogenic granuloma	AF
Mckelvy and Cherrick/1976 [15]	Male/17 years old	Interdental gingiva between lower left two premolars	Not described	AFD
Godjesk et al./1980 [16]	Male/3 years old	Lingual gingiva between lower primary left lateral incisor and canine	Not described	AFD
Chen et al./2006 [17]	Female/2.5 years old	Labial gingiva between upper primary central incisors	Benign tumor of soft tissue origin	AFD
Minamizato et al./2014 [18]	Female/51 years old	Upper left interdental papilla between second premolar and first molar	Benign tumor of gingiva	AFD
Reibel et al./2011 [19]	Female/8 years old	Palatal gingiva between upper right central and lateral incisors	Not described	AFO
This case/2014	Female/3 years old	Facial gingiva of primary upper right central incisor	Pyogenic granuloma	AFO

## References

[1] Takeda Y, Tomich CE (2005). Ameloblastic fibro-odontoma. *Pathology and Genetics of Head and Neck Tumors*.

[2] de Riu G, Meloni SM, Contini M, Tullio A (2010). Ameloblastic fibro-odontoma: case report and review of the literature. *Journal of Cranio-Maxillofacial Surgery*.

[3] Buchner A, Kaffe I, Vered M (2013). Clinical and radiological profile of ameloblastic fibro- odontoma : an update on an uncommon odontogenic tumor based on a critical analysis of 114 cases. *Head and Neck Pathology*.

[4] Buchner A (1989). Peripheral odontogenic fibroma. Report of 5 cases. *Journal of Cranio-Maxillo-Facial Surgery*.

[5] Kusama K, Masahiko M, Moro I (1998). Peripheral ameloblastic fibroma of the mandible: report of a case. *Journal of Oral and Maxillofacial Surgery*.

[6] Abughazaleh K, Andrus KM, Katsnelson A, White DK (2008). Peripheral ameloblastic fibroma of the maxilla: report of a case and review of the literature. *Oral Surgery, Oral Medicine, Oral Pathology, Oral Radiology and Endodontology*.

[7] Nodit L, Barnes L, Childers E, Finkelstein S, Swalsky P, Hunt J (2004). Allelic loss of tumor suppressor genes in ameloblastic tumors. *Modern Pathology*.

[8] Galvão CF, Gomes CC, Diniz MG (2012). Loss of heterozygosity (LOH) in tumour suppressor genes in benign and malignant mixed odontogenic tumours. *Journal of Oral Pathology and Medicine*.

[9] Papagerakis P, Peuchmaur M, Hotton D (1999). Aberrant gene expression in epithelial cells of mixed odontogenic tumors. *Journal of Dental Research*.

[10] Yagishita H, Taya Y, Kanri Y (2001). The secretion of amelogenins is associated with the induction of enamel and dentinoid in an ameloblastic fibro-odontoma. *Journal of Oral Pathology and Medicine*.

[11] Miyake T, Tanaka Y, Kato K (2006). Gene mutation analysis and immunohistochemical study of beta-catenin in odontogenic tumors. *Pathology International*.

[12] Tanaka A, Okamoto M, Yoshizawa D (2007). Presence of ghost cells and the Wnt signaling pathway in odontomas. *Journal of Oral Pathology and Medicine*.

[13] Sathi GA, Tamamura R, Tsujigiwa H (2012). Analysis of immunoexpression of common cancer stem cell markers in ameloblastoma. *Experimental and Therapeutic Medicine*.

[14] Newman PJ (1994). The role of PECAM-1 in vascular cell biology. *Annals of the New York Academy of Sciences*.

[15] McKelvy BD, Cherrick HM (1976). Peripheral ameloblastic fibrodentinoma. *Journal of Oral Surgery*.

[16] Godjesk JE, Dolinsky HB, Schneider LC, Doyle JL (1980). Ameloblastic fibro-dentinoma in the gingiva: report of a case. *Journal of Oral Medicine*.

[17] Chen H, Wang W, Lin Y, Chen Y, Lin L (2006). Gingival ameloblastic fibro-dentinoma-Report of a case in a child. *International Journal of Pediatric Otorhinolaryngology Extra*.

[18] Minamizato T, I T, Ikeda H, Fujita S, Asahina I (2014). Peripheral-type ameloblastic fibrodentinoma with features of so-called “immature dentinoma”. *Oral Surgery, Oral Medicine, Oral Pathology and Oral Radiology*.

[19] Reibel J, Grønbæk AB, Poulsen S (2011). Peripheral ameloblastic fibro-odontoma or peripheral developing complex odontoma: report of a case. *International Journal of Paediatric Dentistry*.

[20] Takeda Y (1999). Ameloblastic fibroma and related lesions: current pathologic concept. *Oral Oncology*.

[21] Kfir Y, Buchner A, Hansen LS (1980). Reactive lesions of the gingiva. A clinicopathological study of 741 cases. *Journal of Periodontology*.

[22] Buchner A, Shnaiderman-Shapiro A, Vered M (2010). Pediatric localized reactive gingival lesions: a retrospective study from israel. *Pediatric Dentistry*.

[23] Yuan K, Chen C, Lin MT (2003). Enamel matrix derivative exhibits angiogenic effect in vitro and in a murine model. *Journal of Clinical Periodontology*.

[24] Keysar SB, Jimeno A (2010). More than markers: biological significance of cancer stem cell-defining molecules. *Molecular Cancer Therapeutics*.

[25] Hoffmeyer K, Raggioli A, Rudloff S (2012). Wnt/*β*-catenin signaling regulates telomerase in stem cells and cancer cells. *Science*.

[26] Järvinen E, Salazar-Ciudad I, Birchmeier W, Taketo MM, Jernvall J, Thesleff I (2006). Continuous tooth generation in mouse is induced by activated epithelial Wnt/*β*-catenin signaling. *Proceedings of the National Academy of Sciences of the United States of America*.

